# Successful resection of malignant hemangiopericytoma originating from left neck and involving superior vena cava

**DOI:** 10.1186/1749-8090-8-S1-P32

**Published:** 2013-09-11

**Authors:** T Minchev, E Manolov, V Marinchev, I Todorov, V Georgiev, I Stoimenov, G Kirova, H Mavrov

**Affiliations:** 1 Thoracic Surgery, Tokuda Hospital, Sofia, Bulgaria; 2 Cardiac Surgery, Tokuda Hospital, Sofia, Bulgaria; 3 ICU, Tokuda Hospital, Sofia, Bulgaria; 4 Medical Imaging Department, Tokuda Hospital, Sofia, Bulgaria

## Background and methods

We report a case of a giant malignant hemangiopericytoma in the left neck and involving and growing in left internal jugular, left subclvian, left brachiocephalic vein, superior vena cava (SVC) and occupying part of the right atrium. Tumor was causing severe dyspnea, dysphagia and initial SVC syndrome. A 77-year-old male was admitted to our hospital due to progressive dyspnea and dysphagia. Physical examination present large tumor mass in left supraclavicular and neck region with listening a trill. CT scan explain a large tumor mass started in level of hyoid in left side neck that involve deep veins and expand vessels. Tumor have a arterial blood circulation in SVC. Transthoracic echocardiography showed a mass occupying a space of the right atrium originating from the SVC. Cervicosternotomy used for surgical access. After bluntly removed the tumor from the left neck and ligation of the jugular and subclavian vein proceed to the vascular reconstruction. SVC reconstruction was performed between the right brachiocephalic vein and the right atrial appendage with ringed pericardial graft. Atrial part of tumor was shoved into a SVC. Tumor with vena cava was removed after reconstruction.

## Results

The postoperative course was uneventful. Chest tube removed in 3^rd^ postoperative day. No evidence of local recurrence and metastasis was obtained in this patient after the operation for a period of 3 years. Additional therapies including radiation and chemotherapy might be considered in our case.

## Conclusion

The management for malignant hemangiopericytomas is not yet standardized because numbers of case are limited. Surgical resection might be done first of all if it is possible. Radiation therapy is generally ineffective to reduce tumor volume. Trial of chemotherapy is controversial. Further accumulation of cases of malignant hemangiopericytoma is necessary to understand pathological mechanisms of the disease and to determine the priority of the possible therapies.

